# Keeping an Open Mind: Cognitive Bias in the Evaluation of an Infant with Posterior-Lateral Rib Fractures

**DOI:** 10.1155/2017/5163094

**Published:** 2017-10-26

**Authors:** Katie Johnson, Donald Chris Derauf, Raymond Stetson, Paul Galardy, Jason Homme

**Affiliations:** ^1^Department of Pediatric and Adolescent Medicine, Mayo Clinic, Rochester, MN, USA; ^2^Department of Pediatric Hematology/Oncology, Mayo Clinic, Rochester, MN, USA

## Abstract

A four-month-old former premature male is incidentally found to have posterior-lateral rib fractures during evaluation of a febrile illness. This finding led to the initiation of a workup for nonaccidental trauma. A thorough history and physical exam ultimately led to the diagnosis, which was not related to abuse. This case highlights a rare sequela of patent ductus arteriosus repair, cautions medical teams to remain aware of how cognitive bias can affect diagnostic decision-making, and emphasizes the importance of a thorough history, physical exam, and medical record review in cases of suspected nonaccidental trauma.

## 1. Introduction

Posterior rib fractures in infants are classically associated with nonaccidental trauma (NAT) [1–5]. This is attributable to both their location and the developmental stage of infants. Posterior rib fractures typically result from a circumferential squeezing of the infant, which creates a levered force at the articulation of the posterior rib and the vertebral body [6]. It would be unusual to sustain a posterior rib fracture from an accidental mechanism, especially for a child who is nonambulatory. Thus, when pediatricians hear the term “posterior rib fracture,” they appropriately place NAT near the top of their differential diagnosis.

This cognitive leap is an example of a heuristic—a diagnostic shortcut that uses clinical acumen to form an ingrained association between a presenting sign or symptom and a likely diagnosis [5, 7]. Other examples of heuristics in pediatrics include the associations between barky cough and croup, slapped-cheek rash and parvovirus, and thumbprint sign and epiglottitis.

While heuristics add efficiency to the diagnostic process, they are not infallible. Heuristics must be taken into consideration with each individual patient context. For example, other causes of rib fractures in infancy include high-impact trauma [8, 9], cardiopulmonary resuscitation [10, 11], thoracic surgery [12], birth trauma [13], and even chest physiotherapy [14]. Underlying conditions such as osteogenesis imperfecta, osteopenia of prematurity [15], and rickets [16] must also be considered since they place certain infants at higher risk of fractures than others [9].

The case to follow is that of a former premature male infant incidentally found to have posterior-lateral rib fractures on chest X-ray (CXR) during evaluation of a febrile illness and how a thorough history and physical exam ultimately led to a diagnosis that was not related to abuse.

## 2. Case Presentation

The patient was a four-month-old former 28-and-5/7-week gestation premature twin male admitted directly from an outside facility for further evaluation of fever, increased sleepiness, and new oxygen requirement.

The patient had a history of prolonged course in the neonatal intensive care unit (NICU) and had been discharged approximately two months prior. Upon presentation to an outside emergency department (ED), he had a two-day history of fever and sleepiness and was noted to have oxygen desaturations that prompted the administration of supplemental oxygen. Laboratory studies were notable for elevated CRP at 116.4 mg/L (normal ≤ 4.9 mg/L), mild thrombocytosis at 494 × 10^3^/mcl (normal 150–350 × 10^3^/mcl), and normal leukocyte count at 12.9 × 10^3^/mcl (normal 5.0–15.0 × 10^3^/mcl).

A CXR was obtained and reviewed by the local treating physician, a third-party teleradiology company contracted by the referring hospital during nonbusiness hours, and the senior pediatric resident at the receiving institution. Consistent between the reports were the absence of pulmonary consolidation (i.e., pneumonia) and the presence of radiopaque patent ductus arteriosus (PDA) clips over the mediastinum. The patient was transferred by ambulance to the receiving hospital.

On admission, the patient was managed with empiric antibiotics, pending the results of blood and urine cultures. Both cultures returned negative for growth. Tests for respiratory syncytial virus and influenza were negative. Pain was managed with scheduled Tylenol, and oxygen support was gradually weaned over the next eight hours.

**Figure 1:**
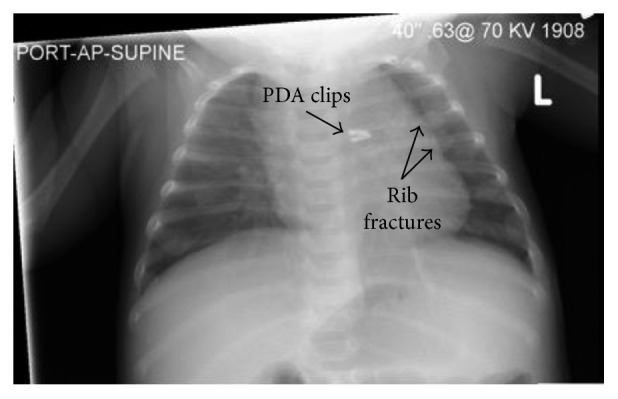
Admission chest X-ray.

The morning following admission, the referring physician contacted the primary team to inform them that a staff radiologist had reviewed the CXR and amended the initial report, indicating “there is callus associated with the posterior-lateral left fourth and fifth ribs, sequela of healing/healed fractures. There is no finding suggestive of acute osseous abnormality or pneumothorax” (Figure 1). These findings were confirmed by the staff radiologist at the receiving institution. Review of the patient's medical record indicated that the rib fractures were a new finding compared to the most recent CXR, which had been obtained three months priorly, directly after PDA repair in the NICU.

Upon receipt of the outside radiology report, the primary team became concerned for NAT. A skeletal survey was ordered to evaluate for other fractures, and further history and physical exam were obtained. Physical exam revealed two small, well-healed scars on the infant's left upper back, overlying the site of the fractures. There were no other signs of injury such as cutaneous bruising, scratches, burns, oral trauma, or altered mental status.

When the scars were brought to the attention of the patient's father, he suggested that they may be attributable to the patient's PDA repair. This prompted review of the operative report, which read, “a limited left lateral thoracotomy was performed and the hemithorax entered through the third interspace.” The posterior-lateral rib fractures were ultimately attributed to this surgical intervention from PDA repair. The skeletal survey was negative for any other fractures.

## 3. Discussion

This case highlights a rare cause of posterior-lateral rib fractures in infants: surgical PDA repair. There is only one reported case in the literature of rib fractures following percutaneous PDA repair [12], and none reported following surgical PDA repair [17].

PDA repair is a commonly performed procedure, particularly among premature infants. When PDA fails to close naturally or with pharmacologic management, several options are available: surgical repair with posterior-lateral thoracotomy and clip placement or ligation [18]; percutaneous repair (i.e., placement of an occluding device over a wire-guided catheter) [19, 20]; and less commonly, video-assisted closure [18].

In surgical PDA repair, the two ribs above and the two ribs below the entry interspace are retracted in stages using a rib spreader, to limit rib fractures [18]. In the patient presented here, the surgical team entered through the left third interspace, which was directly above the level of the two identified fractures (i.e., left fourth and fifth ribs). It was concluded that the tension produced by retraction of the fourth and fifth ribs was the cause of the rib fractures, possibly compounded by a component of osteopenia of prematurity or metabolic bone disease. Further discussion with the surgical team suggested that the radiographic findings may not be fractures at all, but instead, periosteal reaction from manipulation of the ribs during the procedure.

This is not only the first report of the association between surgical PDA repair and posterior-lateral rib fractures, but also the first report to our knowledge on the diagnostic overlap between history of PDA repair and initiation of a NAT workup. Indeed, one could argue that a more comprehensive NAT workup was warranted in this case, including a social work assessment, head CT, ophthalmologic exam, labs for abdominal injury, and a follow-up skeletal survey in two weeks. In this case, the patient's family had undergone prior social work assessment while in the NICU, and the primary team felt confident that the posterior-lateral rib fractures with overlying surgical scars on exam and corroborative operative report were sufficient to attribute the fractures to surgical etiology. That being said, the importance of a full NAT evaluation when abuse is suspected cannot be overstated.

An important element of this case is the cognitive bias that can accompany the use of heuristics in medicine. Specifically, we demonstrate how the association between posterior rib fractures in an infant and suspicion of NAT can lead to inaccurate diagnosis if not paired with a careful history, physical exam, and medical record review.

While heuristics serve an important purpose in medicine, they can also predispose clinicians to cognitive bias, which may disrupt accurate clinical reasoning. One such bias is anchoring, in which a clinician commits to a diagnosis early in the workup of a patient at the expense of considering other possibilities [7]. In this particular case of a former premature infant with posterior-lateral rib fractures, an open differential diagnosis is of utmost importance, given the complexity of the patient's past medical history, the increased susceptibility of preterm infants to osteopenia and fractures [21–23], and the repercussions of a presumptive diagnosis of NAT. Obtaining further history and a close physical exam helped mitigate the cognitive bias of anchoring to a presumed diagnosis of inflicted trauma.

We present this case as a caution to providers to utilize heuristics carefully, with an understanding of how cognitive bias is inextricably part of the diagnostic process. We hope to impress upon readers the value of a thorough history, physical exam, and medical record review in cases of suspected NAT. Most novel about this case, however, is the association between history of surgical PDA repair and posterior-lateral rib fractures, which had previously not been documented in the literature. This is a rare complication of PDA repair by any method (i.e., surgical or percutaneous), but does occur and should be considered as part of the differential diagnosis for infants with posterior-lateral rib fractures.
